# Investigating Effects of Cold Water Hand Immersion on Selective Attention in Normobaric Hypoxia

**DOI:** 10.3390/ijerph16162859

**Published:** 2019-08-10

**Authors:** Hayden D. Gerhart, Yongsuk Seo, Jung-Hyun Kim, Brittany Followay, Jeremiah Vaughan, Tyler Quinn, John Gunstad, Ellen L. Glickman

**Affiliations:** 1 Department of Kinesiology, Health, and Sport Science, Indiana University of Pennsylvania, Indiana, PA 15705, USA; 2 Centers for Disease Control and Prevention, National Institute for Occupational Safety and Health, National Personal Protective Technology Laboratory, Pittsburgh, PA 15236, USA; 3 Department of Sports Medicine, Kyung Hee University, Yongin-si, Gyenonggi-do 17104, Korea; 4 Department of Exercise Science, Ripon College, Ripon, WI 54971, USA; 5 Human Performance, Sport and Health Department, Bemidji State University, Bemidji, MN 56601, USA; 6 Environmental Physiology Laboratory, Kent State University, Kent, OH 44242, USA

**Keywords:** cold water hand immersion, selective attention, arousal, thermal sensations, normobaric hypoxia

## Abstract

This study investigated the effect of cold-water hand immersion on selective attention as measured by the Stroop Color Word Test in nomorbaric normoxia and hypoxia. Ten healthy men rested for 60 min, after which they immersed their non-dominant hand into 5 °C water for 15 min. The interference score of the Stroop Color Word Test and thermal sensation were measured at baseline in the final 5 min of resting and in the final 5 min of cold water hand immersion. The interference score was not influenced by hypoxia but was found to be significantly improved compared to resting in both conditions during cold water hand immersion. Selective attention improved during 15 min of cold-water hand immersion, with increased thermal sensations rated as “very cool” of the immersed arm. Cold-water hand immersion may be helpful in improving cognitive function in normoxia and normobaric hypoxia.

## 1. Introduction

Cognitive function plays an important role in daily life, optimizing performance and safety. However, cognitive function is often altered during exposure to both normobaric hypoxia (a state of reduced oxygen at normal atmospheric pressure) and hypobaric hypoxia (a state of reduced partial pressure of oxygen due to lower barometric pressure) [1,2,3]. This is of significant importance for tactical, military, and first responder populations who are expected to operate optimally in various environmental conditions. Normobaric hypoxia can be induced by intrinsic factors (defective delivery or utilization of oxygen) [4] and extrinsic factors (carbon monoxide inhalation, etc.) [5,6]. Specifically, a study involving chronic hypoxic exposure in rats led to significantly impaired systolic and diastolic function. Additionally, the chronic hypoxic exposure led to a significantly reduced oxygen uptake, which eludes to similar responses in humans [4]. A common extrinsic factor, carbon monoxide inhalation, can severely impact all body systems, most specifically the cardiovascular system and central nervous system. The increased tissue hypoxia at the onset of carbon monoxide inhalation can lead to a wide array of negative symptoms, including dizziness, nausea, and potentially seizures in all age groups [5]. Cognitive impairment is influenced by the magnitude of arterial oxygen saturation due to lack of oxygen availability [7]. However, it has been suggested that both acute exercise and transcranial laser stimulation increase mitochondrial respiration and brain-derived neurotrophic factor [8,9,10], indicating that both stimulations activate the prefrontal cortex and improve the prefrontal-dependent cognitive function (i.e., executive function including sustained attention, response inhibition, working memory) [11,12,13].

Although aforementioned strategies (acute submaximal exercise and laser transcranial stimulation) are well established for improving cognitive performance, utilization of these strategies may not be beneficial because of skeletal muscle fatigue by exercise and device settings. Another stimulus that has been found to improve cognitive function is the cold pressor stress (CPS) [14]. The association between CPS and SCWT has been shown to be both positive and inverse depending on stimulus duration and intensity [15,16]. A previous study reported that perception of acute pain by CPS is associated with executive function as measured by the Stroop Color Word Test (SCWT) within a short period of time [15,17]. Further, CPS has been found to facilitate eye blink conditioning (a classical test widely used to examine learning and memory) and spatial navigation performance in healthy men after only one minute of hand exposure [14]. However, a recent meta-analytical review of eight studies concluded that experimentally induced pain sensation by CPS produces an effect on attention but not on executive function [18].

Despite evidence that CPS induces increased arousal [19] which enhances cognitive function, the effect of longer duration cold water hand immersion (CWHI) on cognitive function remains unclear. Therefore, the purpose of the present study was to investigate the effect of CWHI on executive function in normobaric hypoxia. It was hypothesized that the autonomic nervous system would be stimulated by immersion of the hand in cold-water, which would decrease finger temperature, thus improving selective attention in normobaric hypoxia and increasing SCWT performance.

## 2. Materials and Methods

The Institutional Review Board of Kent State University (IRB #: 16-096) approved this study and all participants signed a consent form prior to participation. Each of the experimental sessions (normobaric 13% O_2_ and 21% O_2_) was counterbalanced and blinded to the participants, separated by at least seven days. The 13% O_2_ was selected based on previous studies indicating the impairments of cognitive function and mood state [12,13] and the O_2_ content found at Pikes Peak, a popular summit found in Colorado where altitude research is prominent.

### 2.1. Participants

Ten healthy nonsmoking men (age: 23 ± 3 years, height: 179 ± 5 cm, weight: 83 ± 9 kg, BMI: 26 ± 3 kg/m^2^) volunteered for this study. Participants were recreationally active and free of any cardiovascular, pulmonary, or metabolic diseases. Participants were excluded if they had reported previously experiencing frostbite, Raynaud’s disease, sickle cell anemia, or any other condition or medication that affected circulation or other cardiovascular variables. All subjects reported that they had never experienced loss of consciousness at altitude or normobaric hypoxia within two months prior to participation.

### 2.2. Procedures

Prior to participation, participants underwent prescreening with a medical history questionnaire and were informed of the protocol for each experimental session. Participants were instructed to abstain from food for 3 h to stabilize substrate utilization, as well as strenuous exercise and alcohol for the previous 24 h. Following prescreening, participants were familiarized with the study protocol and instrumentation including SCWT in order to minimize the learning effect on the day of testing. The number of practice sessions varied between participants; once performance plateaued, familiarization sessions were discontinued.

On days of experimental testing, participants dressed in t-shirts and shorts. Baseline measurements of heart rate (HR), stroke volume (SV), blood pressure (BP), peripheral oxygen saturation (SpO_2_), finger temperature (Tf), and SCWT were obtained while participants sat quietly on a chair outside of the hypoxic chamber for approximately 30 min in a thermoneutral condition (25 °C, 40% relative humidity [RH]). Following baseline measurements, participants moved into a normobaric hypoxia chamber (Colorado Altitude Training, Louisville, CO) for a 60-min resting period to establish stable oxygen saturation. The condition within the hypoxia chamber was unknown to participants, randomized, and was counterbalanced throughout the study (either 13% O_2_ or 21% O_2_). During the final 5 min of the resting period, HR, SV, BP, SpO_2_, Tf, and SCWT were assessed a second time. Following resting, participants immersed their non-dominant hand into 5 °C water for 15 min. All variables were assessed a third time during the final 5 min of 15 min of water immersion. The 15 min of cold water hand immersion was chosen to control and stabilize finger temperature across all participants, subsequently, sympathetic nervous system and arousal. A minimum of 48 h and maximum of 72 h between experimental sessions was implemented throughout the study.

### 2.3. Instrumentation

A skin thermistor (ITP082-25, Nikkiso-Therm Co., Ltd., Japan) was affixed to the non-dominant middle finger with a transparent dressing film (Tegaderm, 3M, St. Paul, MN) to measure finger skin temperature (proximal nail fold). HR, SV and BP were obtained by the use of a BioZ-Dx, which measures changes in thoracic blood flow impedance using a high-frequency (60-kHz minimum, low-amplitude, 4.0-mA rms maximum) alternating electrical current (Cardio dynamics, San Diego, CA). SpO2 was assessed through the use of a pulse oximeter (Oxy-Go, Roslyn, NY) placed onto the dominant hand ring finger, which detects oxygenated hemoglobin through the absorption of specific wavelengths emitted by the oximeter.

Thermal sensation of the immersed arm was measured at baseline, following resting, and at the end of CWHI using a 0–10 scale (0 = Nothing at all, 5 = Cold, and 10 = Very Very Cold) [20].

Cognitive function tests were assessed with Automated Neuropsychological Assessment Metrics-4th Edition (ANAM4), a computerized cognitive test battery first developed by the Department of Defense with subtests designed to assess a variety of cognitive domains. The specific subtest utilized for this study was the SCWT.

SCWT assesses processing speed, selective attention, interference score, and executive functioning. The SCWT consists of three 45-second tests. The first test (word: W) involves pressing a corresponding key for each black-printed word (1 for red, 2 for green, 3 for blue). The next test (color: C) requires pressing the corresponding key based on color. A series of colors including red, green or blue are presented on the screen. In the final test (color-word: CW), a series of words (red, green, blue) are presented in a color that does not match the name of the color displayed by the word. The participants are required to press the response key assigned to color. The interference score of SCWT was automatically calculated as follows: interference score = CW − [(W × C) / (W + C)]. A higher score is indicative of better cognitive performance.

### 2.4. Data Analysis

All statistical analyses were conducted using statistical software package (SPSS v.19.0, IBM, Armonk, NY, USA). For SCWT, two-way (two conditions by three time points) repeated measure analyses of variance (ANOVA) was used. Additionally, two-way (two conditions by six time points) repeated measure analyses of variance (ANOVA) was used for SpO_2_, Tf, HR, SV, and MAP measurements. When ANOVA indicated a significant interaction or main effect, a post-hoc paired sample *t*-test was performed to determine where those differences existed. Statistical significance was set at *p* < 0.05 and all data are presented as mean ± standard deviation (SD).

## 3. Results

### 3.1. Stroop Color Word Test and Thermal Sensation

**Figure 1:**
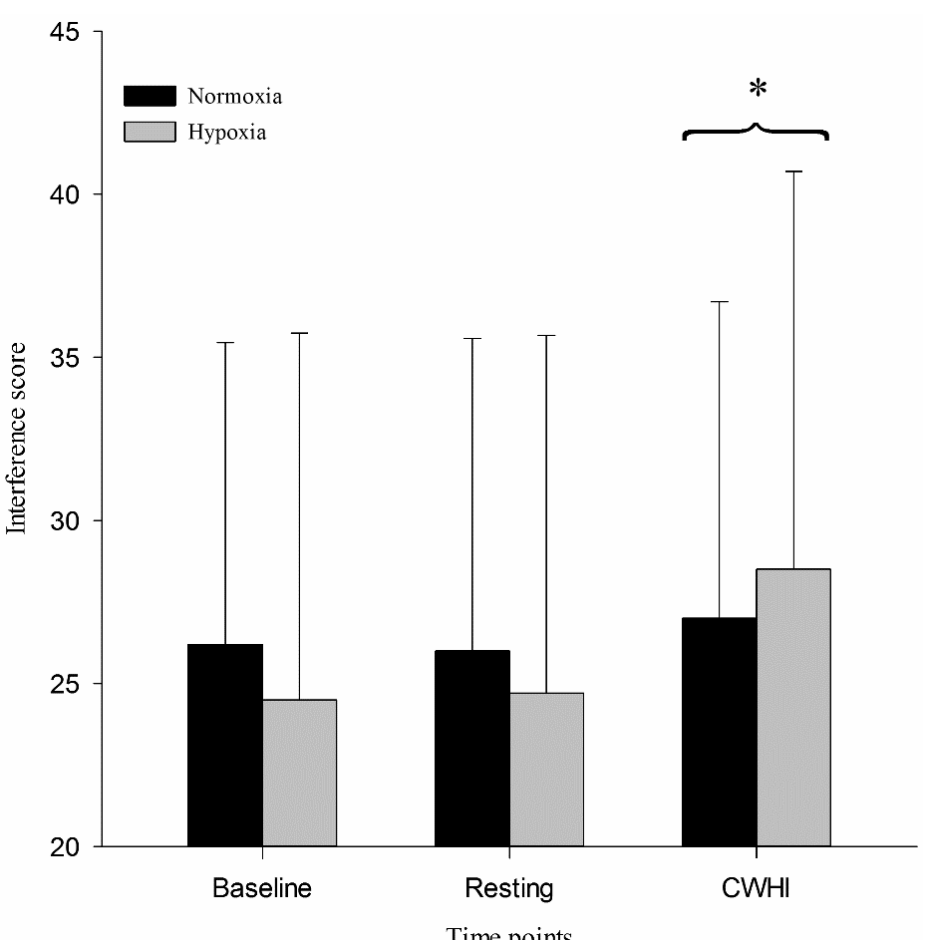
Interference score of the Stroop Color Word Test during baseline, resting, and CWHI. * *p* < 0.05, significantly different compared to “Resting”.

The interference score of the SCWT demonstrated a main effect for time (F = 3.739, *p* = 0.044, power = 0.607), but no main effect for condition (F = 0.194, *p* = 0.670, power = 0.068) and no interaction (F = 1.387, *p* = 0.275, power = 0.259). Post-hoc analysis revealed that the interference score was not influenced by hypoxia (*p* = 1.000) but, during CWHI, it was found to be significantly improved compared to 60-min resting (Figure 1). Thermal sensation of the immersed arm was rated as significantly “very cool” during CWHI (normoxia: 1.5 ± 0.8, hypoxia: 1.4 ± 0.6) compared to baseline (normoxia: 0.1 ± 0.2, hypoxia: 0.0 ± 0.0; *p* ≤ 0.001), and resting (normoxia: 0.0 ± 0.0, hypoxia: 0.0 ± 0.0; *p* ≤ 0.001).

### 3.2. Peripheral Oxygensaturation and Finger Temperature

**Figure 2:**
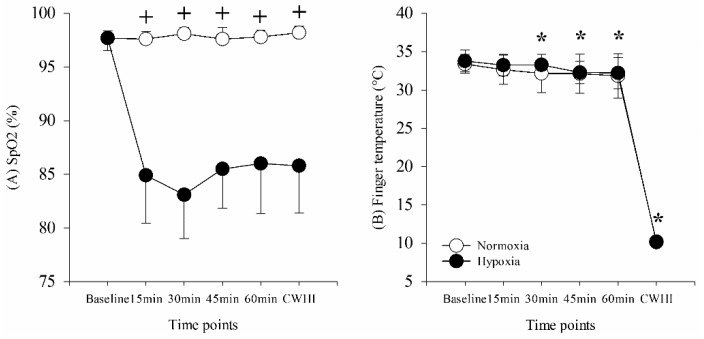
(**A**) Peripheral oxygen saturation (SpO_2_) and (**B**) finger temperature (Tf) across time points. ^+^ *p* < 0.05, significantly different from hypoxia; * *p* < 0.05, significantly different from baseline.

SpO_2_ demonstrated a main effect for time (F = 29.279, *p* ≤ 0.001), main effect for condition (F = 136.0, *p* ≤ 0.001), and interaction (F = 34.855, *p* ≤ 0.001). SpO_2_ was significantly decreased following exposure to hypoxia (*p* ≤ 0.001) and remained so through the trial (Figure 2A). Finger skin temperature (Tf) demonstrated a main effect for time (F = 1068.358, *p* ≤ 0.001), but no main effect for condition (F = 1.291, *p* = 0.289, power = 0.171) and no interaction (F = 1.011, *p* = 0.424, power = 0.322). Tf was gradually decreased during the 60 min after exposure to hypoxia and was significantly decreased during CWHI (Figure 2B).

### 3.3. Cardiovascular Function

**Figure 3:**
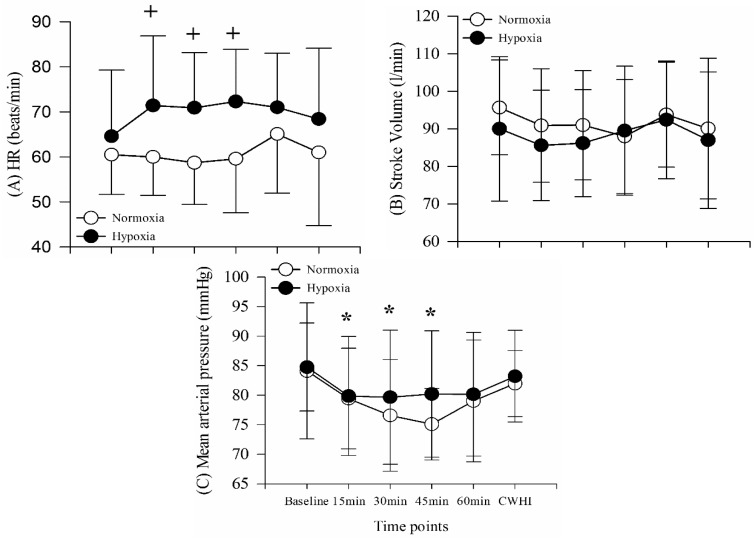
(**A**) Heart rate (HR), (**B**) stroke volume, and (**C**) mean arterial pressure across time points. ^+^ *p* < 0.05, significantly different from hypoxia; * *p* < 0.05, significantly different from baseline.

Heart rate demonstrated no main effect for time (F = 2.175, *p* = 0.074, power = 0.659), but a main effect for condition (F = 11.96, *p* = 0.007, power = 0.867) and no interaction (F = 1.831, *p* = 0.126, power = 0.572). HR was significantly higher during hypoxia than normoxia at 15 (*p* = 0.017), 30 (*p* = 0.001), and 45 min (*p* = 0.001) (Figure 3A). Stroke volume indicated no main effect for time (F = 1.156, *p* = 0.345, power = 0.372), no main effect for condition (F = 4.952, *p* = 0.053, power = 0.511), and no interaction (F = 0.564, *p* = 0.727, power = 0.189) (Figure 3). Mean arterial pressure (MAP) indicated a main effect for time (F = 4.396, *p* = 0.002), no main effect for condition (F = 0.797, *p* = 0.395, power = 0.126), and no interaction (F = 0.840, *p* = 0.529, power = 0.273). MAP was significantly decreased following exposure to hypoxia, but returned to baseline levels during CWHI (Figure 3C).

## 4. Discussion

The purpose of this study was to quantify executive function during CWHI in normobaric normoxia and hypoxia. We hypothesized that selective attention as measured by SCWT would be improved during 15 min of CWHI because of increased sympathetic nervous system activation and arousal. This hypothesis was supported in the present study in that SCWT was improved during CWHI compared to resting both in normoxia and hypoxia. This study also shows that finger temperature decreased during CWHI, which stimulated significantly increased thermal sensation ratings toward “very cool” during CWHI, although no significant cardiovascular changes were shown during 15 min of CWHI.

### 4.1. Pain Sensitivity and Cognitive Function

It was previously reported that short duration CWHI (maximum of 120 s) shows an association between pain sensitivity and cognitive inhibition [15,17]. While the SCWT reflects cognitive inhibition ability, cognitive inhibition is also a crucial factor in determining pain sensitivity [17]. While this study did not measure pain sensitivity, a subjective measurement of thermal sensations was taken. This study showed significant changes towards a “very cool” rating in thermal sensation among both conditions during CWHI compared to resting, in conjunction with increased SCWT scores. It can be surmised that a similar relationship exists between thermal sensation and cognitive inhibition. CWHI has been previously shown to increase sympathetic nervous system responses and an increase in hypothalamic–pituitary–adrenal (HPA) axis activity [21,22]. In the current study, improved cognitive function was displayed through increased SCWT performance, which might be modulated via these increases in the sympathetic nervous system and HPA axis activity from the increased thermal sensation measured subjectively during CWHI.

### 4.2. Arousal

It is also important to consider the effect that arousal may have on SCWT in this current investigation. Cold pressor stimulation has also been previously shown to induce increased arousal [19]. It has been previously shown that exposure to the cold induces increased SCWT interference scores, supporting the cold arousal effect theory [23,24]. In the current investigation, increased arousal from CWHI was likely maintained throughout the 15 min and contributed to the increased SCWT performance in both the hypoxia and the normoxia condition. Furthermore, a previous study showed that immersion time up to 120 s was positively associated with the SCWT interference score, indicating that a longer immersion time is associated with a higher score and improved cognitive function [17]. The current study showed that an increased SCWT performance during 15 min of CWHI is in agreement with previous studies and expands on the previous conclusions [17]. While a positive relationship was found previously between immersion time and the SCWT interference score, it remains unclear if this relationship would be maintained for longer than 120 s of immersion. It seems that even during 15 min of immersion, cognitive inhibition is increased resulting in increased SCWT performance

### 4.3. Cardiovasculat Response

The current study shows no cardiovascular responses during 15 min of CWHI with HR, SV, and BP remaining unchanged from 60 min resting in either hypoxia or normoxia. Previous research has shown a similar stable BP response during cold water immersion of the right hand in 5 °C water for 30 min [25]. However, Sendowski, Savourey, Besnard and Bittel [25] showed a slight increase in heart rate immediately after immersion followed by a decrease to the baseline level two minutes following immersion. Furthermore, they showed a slight decrease in HR throughout the 30 min of CHWI, resulting in a 10 beat decrease from baseline [25]. The contradictory cardiovascular response to CWHI shown in the current study can likely be attributed to shortened hand immersion time. The current study used only 15 min of CWHI which may not be a long enough duration in 5 °C water to show significant cardiovascular changes such as decreased HR. This notion is further confirmed by the fact that significantly decreased HR was not detected until minute 18, following immersion [25]. Additionally, previously shown increases in HR and BP in the minutes immediately following immersion of the hand are not presented in this data as cardiovascular variables were measured only at the beginning and end of hand immersion. Finally, it must be considered that a recent review found inconsistent cardiovascular responses to cold pressor stimulation, making the results of the current study not surprising given the methodological differences previously mentioned [19].

This study included several limitations that should be noted. First, the measurement of cardiovascular variables was not done continuously, which limited the study’s ability to detect changes in cardiovascular variables throughout CWHI as seen in previous research. Additionally, only a single duration of CWHI was used, which limited this study’s ability to detect differences in responses that may be duration dependent. Lastly, a direct measure of pain sensitivity, thermal sensation scores, shifting towards “very cool” during CWHI have indicated increased SCWT scores. However, this relationship requires further investigation into causality.

Several strengths of this study should also be mentioned. The current study provided a novel examination of CWHI in hypoxia and normoxia. Additionally, this study used longer duration CWHI than typically used, which allowed for unique interpretations of subjective, cardiovascular, and cognitive responses to this extended stimulus. While the current literature, including this study, has shown increased cognitive inhibition and SCWT performance with CWHI, further investigation must be conducted to determine both lower and upper thresholds that may exist for this effect. Determining minimum and maximum time of CWHI to improve cognition would be useful information when working to apply this method in less controlled occupational or recreational hypoxic environments.

## 5. Conclusions

SCWT performance was improved during CWHI compared to resting in both conditions. This result may have been modulated by increased sympathetic nervous activity in response to decreased finger temperature and increased ratings of thermal sensation during CWHI. While future research is needed to demonstrate causality, CWHI may be a simple and effective way to improve cognitive function during occupational and recreational tasks in normoxia or hypoxia. This is especially important in settings where cognition is potentially compromised and cognitive performance is paramount to occupational safety and task execution such as in military or first responder operations.
